# Understanding Connections between Nature and Stress among Conservation-Engaged Adolescents Using Photovoice Methodology

**DOI:** 10.3390/ijerph20054280

**Published:** 2023-02-28

**Authors:** Kim Hartley, Jonelle Prideaux, Lisa M. Vaughn

**Affiliations:** 1 Cincinnati Children’s Hospital Medical Center, 3333 Burnet Avenue, Cincinnati, OH 45229, USA; 2 Department of Pediatrics, College of Medicine, University of Cincinnati, 3230 Eden Ave, Cincinnati, OH 45267, USA

**Keywords:** greenness, greenspace, children, teen, youth, pediatric, photovoice, stress, mental health, qualitative participatory research

## Abstract

While the literature supports positive associations between nature and adolescent mental health, mechanisms are not well understood, and assessment of nature varies widely among existing studies. To partner with the most insightful informants, we enrolled eight adolescent participants from a conservation-informed summer volunteer program, applying qualitative photovoice methodology to understand their use of nature to relieve stress. Across five group sessions, participants identified four themes: (1) Nature shows us different aspects of beauty; (2) nature helps us relieve stressful experiences by balancing our senses; (3) nature gives us space to find solutions; and (4) we want to find time to enjoy nature. At the conclusion of the project, youth participants reported that the research experience was overwhelmingly positive, enlightening, and inspired appreciation of nature. We found that, while our participants unanimously reported that nature relieved their stress, prior to this project, they were not always intentional in seeking time in nature for this purpose. Through the photovoice process, these participants noted the usefulness of nature for stress relief. We conclude with recommendations for leveraging nature to decrease adolescent stress. Our findings are relevant for families, educators, students, healthcare professionals, and anyone who works with or cares for adolescents.

## 1. Introduction

Adolescents have recently reported feeling extraordinary stress due to political events, social unrest, and the global COVID-19 pandemic [1]. Although stress can be an appropriate reaction to perceived harmful stimuli, it becomes problematic when stress is prolonged without periods of relief, potentially leading to an array of physical and mental health disorders [2] that may extend into young adulthood and beyond. Adolescence is a critical developmental period during which chronic stress increases due to a combination of changes in the brain, social pressures, and emergence of mental health concerns [2]. There is an urgent need to equip adolescents with tools to relieve stress, particularly in the context of the drastic rise in adolescent mental health concerns since the COVID-19 pandemic. These efforts may have lifelong benefits.

Exposure to nature is increasingly prescribed for improving mental health in adolescence [3,4]. Nature may be generally defined as trees, flowers, plants, and grasses and is contrasted with built or man-made surroundings. In epidemiologic studies, exposure to nature may be quantified using land use maps or satellite imagery to objectively measure variables such as distance to parks, surrounding vegetation, and tree counts, while some studies use subjective measures such as reported distance to parks or time spent in nature [4]. Recent evidence supporting a positive association between exposure to nature and decreased stress in adolescents includes increased recovery from stress among high school students who had a view of nature from their classroom window [5], decreased psychological stress associated with seeking exposure to greenness among an urban adolescent population [6], decreased serious psychological distress with increased greenness around the homes of adolescents [7], and a literature review finding exposure to nature contributing to a general reduction in the physiological symptoms of stress [8]. One study found beneficial longitudinal relationships between exposure to nature and reduction of psychosocial indicators of stress in adolescence [9].

Nature’s health benefits are proposed to occur through three mechanisms: restoring capacities via relaxation and mental restoration, building capacities via physical activity and socialization, and reducing harm via improvements to the environment such as removing air pollutants and reducing extreme heat [10]. However, it is unclear which of these mechanisms may apply for adolescent stress relief because there is a gap in our understanding of the motivations and barriers for adolescents using nature to relieve stress. Additionally, current methods of quantifying nature for examining associations with health rely heavily on remote sensing data, such as the Normalized Difference Vegetation Index (NDVI) or Enhanced Vegetation Index (EVI), which use satellite imagery to quantify the amount of chlorophyll reflected by leaves and grasses around a geocoded point. However, these indices are criticized for their inability to characterize quality or accessibility of greenspace [11] and may not accurately capture adolescents’ preferences for, or use of, nature to relieve stress. This inhibits the understanding that is critical for informing future research and interventions to create and protect the natural features that are most meaningful for relieving adolescent stress and for developing educational programming to support adolescents’ use of nature as a tool for relieving their stress.

We began this project with the belief that nature would best relieve stress among adolescents who feel positively toward nature and that positive feelings toward nature would be strongest among adolescents already engaged in conservation activities.

## 2. Materials and Methods

### 2.1. Photovoice Methodology

To obtain data from the most insightful informants, we used photovoice methodology to obtain a nuanced understanding of adolescent perspectives on nature and stress from youth participants in a conservation-informed youth volunteer program. Photovoice is a widely used participatory research methodology in which participants take and discuss photographs of their lived experiences and communities [12,13]. Although photography takes a central role in this method, our goal was to provide space for our participants to voice their experiences [13] rather than to analyze the photos without the context of shared perspectives.

### 2.2. Recruitment and Youth Participants

Consistent with qualitative purposive sampling, whereby participants are enrolled who may best inform the research question and recommendations for sample sizes between six and ten participants when using photovoice methodology, we enrolled eight adolescents to participate in five guided sessions, meeting once a week over the course of 4 weeks and culminating in an exhibition for members of their community. The participants were all affiliated with a local zoo’s summer teen volunteer program designed to build knowledge around animals, horticulture, and conservation; provide leadership experience; and cultivate professional skills [14]. We contacted the 226 adolescent participants of the summer 2022 session via email with information about the research project. Twelve adolescents indicated interest in participating. Four could not commit to the schedule, and eight adolescents were enrolled after obtaining consent from an adult caregiver and assent from the adolescent.

### 2.3. Photovoice Process

Photovoice is a participatory action research strategy for promoting health [13], in which participants use photographic images to prompt discussion around meaningful questions. In this study, photo discussions were facilitated using the SHOWeD method, reconceptualized by Wang, Yi [13] from philosopher and educator Paulo Freire’s pedagogy for stimulating critical thinking though discussion of images, first published in 1968 [15]: What do you **S**ee here? What’s really **H**appening here? How does this relate to **O**ur lives? **W**hy does this problem or this strength exist? What can we **D**o about this?

The photo discussions were intended to explore how these youths experience nature as a tool for managing stress and what limits their access to nature based on two prompts: (1) “What nature is around you (home, school, work, other places you spend time)? How does nature make you feel? What is it about nature that can help relieve your stress?” and (2) “Last week, we talked about how you sometimes use nature to relieve your stress. What might prevent you from doing so?”

We met with youth participants for five sessions over 4 weeks. See Table 1 for a breakdown of the session activities. Session 1 included an overview of the project and an introduction to the photovoice methodology. Sessions 2 and 3 included discussions of the photographs based on the assignments. Session 4 included thematic review and preparation for the final exhibition. Session 5 included the final exhibition where the youth in this project presented the study findings to their community.

### 2.4. Data Collection

In response to each of the prompts, participants were instructed to take approximately 20 photos, from which they were asked to select 1 or 2 photos to share with the group. The number of photos they were encouraged to take greatly exceeded the number they shared with the group to reduce pressure of getting the perfect photo on the first try. Youth were guided through journaling activities at the beginning of both Sessions 2 and 3 to prepare to share and discuss their selected photo(s) among the group.

### 2.5. Data Analysis

In Sessions 2 and 3, participating adolescents engaged in photo discussions based on their chosen selection of a photograph for that week’s assignment to share with the group. Based on SHOWeD questions, each youth took turns sharing their photograph, to which the group was then invited to respond and share their perspectives. Each photo discussion session ended with a brief member-checking discussion of the themes that the researchers noted from the photo discussions.

In Session 4, adult facilitators guided a thematic analysis with the youth to create overarching themes that were derived from the themes identified during Sessions 2 and 3. Once youth agreed upon the themes, they were guided through a two-step process to identify which images best captured each theme. The adolescents voted as a group on which theme they felt best matched each image selected. Although any one image could capture the ideas of more than one theme, the goal was to showcase the images with the most group-level endorsement without overcomplicating the results.

### 2.6. Community Exhibition

In Session 4, adult facilitators guided youth through writing exercises and a group discussion to prepare for their community exhibition. Each adolescent selected a theme to either co-present or individually present in a poster-session style, and all participants wrote at least one comment about the way their theme was captured by the images. The same week, adolescents shared their findings and moderated a question and answer session with their families, invited guests, and zoo leadership at an exhibition held at the partnering zoo (Session 5).

## 3. Results

### 3.1. Participant Characteristics

Eight youth were enrolled as participants in the study. All youth were participants of the Cincinnati Zoo and Botanical Garden ZooTeens program in Summer 2022. Some participants knew each other prior to the study. All were between 14 and 17 years of age. All resided and attended high school in urban or suburban Cincinnati, Ohio. Five participants identified as female and three identified as male.

### 3.2. Nature and Adolescent Stress—Themes

Youth identified and named four salient themes during the sessions:

#### 3.2.1. Theme 1: Nature Shows Us Different Aspects of Beauty

**Figure 1:**
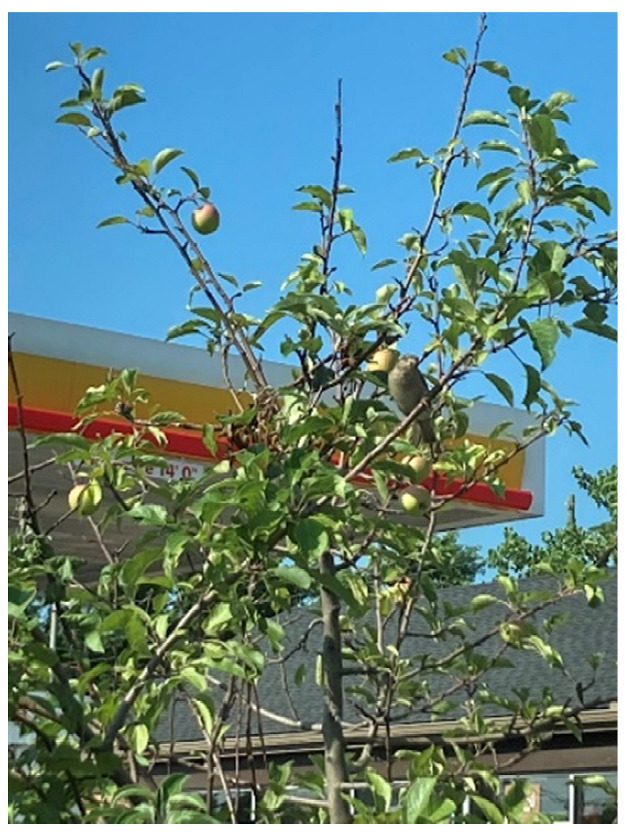
A bird in a pear tree within a developed urban landscape.

**Figure 2:**
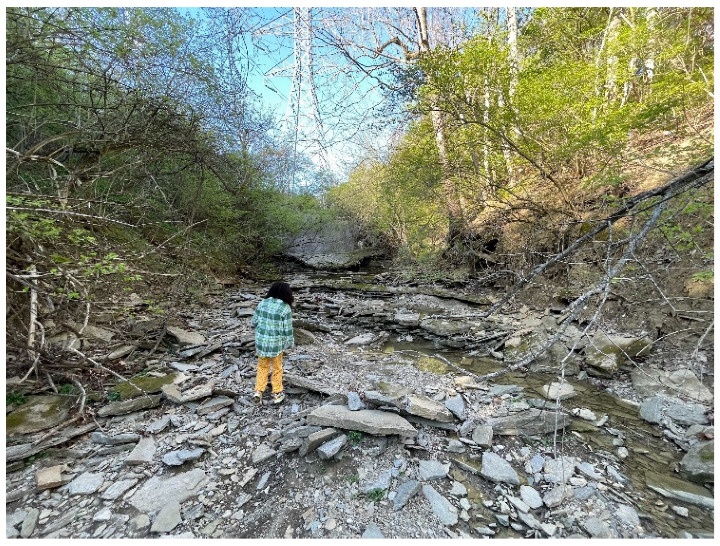
A tower in the background of an otherwise undeveloped area.

In their photos, youth depicted the juxtaposition of natural and man-made elements. For example, in one photo (Figure 1), a bird is seen in a fruit tree next to a gas station, alleviating the stress of a tense car ride. In another (Figure 2), power lines can be seen in an otherwise undeveloped landscape. In both cases, adolescents found unexpected surprises. Some excerpts from youth’s summary statements highlight this concept:

*“Nature gives us unexpected surprises like seeing a rare bird or plant. Nature gives us ways to appreciate surprises.”*

*“Nature shows us things that are beautiful but different than what we would usually think of. Nature shows us different things that help us recognize the different aspects of beauty in the world.”*

#### 3.2.2. Theme 2: Nature Helps Us Relieve Stressful Experiences by Balancing Our Senses

**Figure 3:**
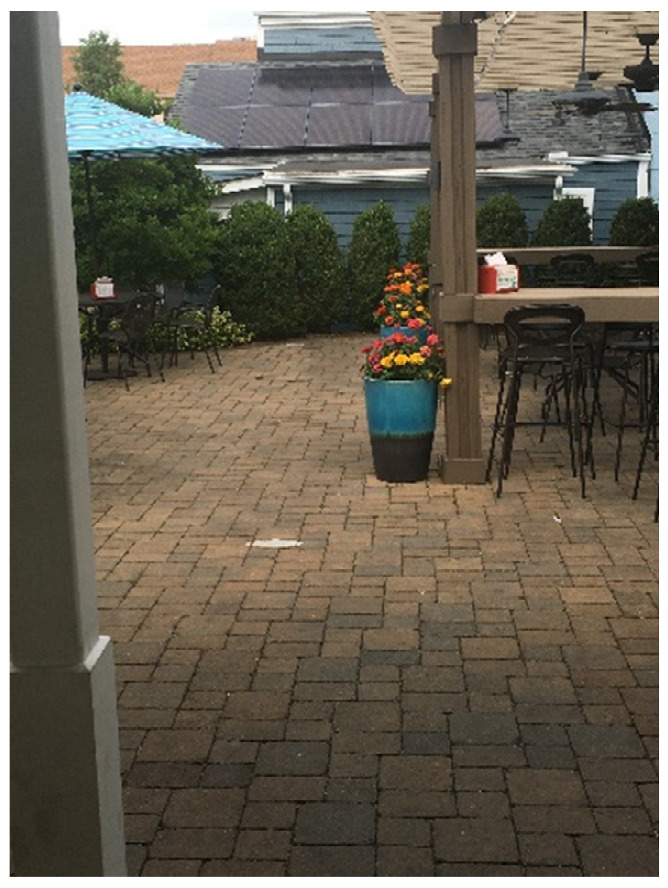
A planter with bright flowers on a paved patio.

Theme 2 highlights the ways that youth felt able to release feelings of overstimulation through exposure to nature. One youth shared a photo with a flowerpot on a patio (Figure 3). He shared that the photo was taken during a particularly stressful work shift in a suburban restaurant, but when he stepped onto the patio during a break, he appreciated the touch of colorful nature there.

*“In our daily lives, we encounter a lot of man-made and unnatural sights, smells, sounds, etc. and it can become very overwhelming. We can become overstimulated and that can inhibit us from thinking clearly and feeing safe and comfortable. Being in nature can calm that overstimulation and allow our brains to rebalance and refocus.”*

**Figure 4:**
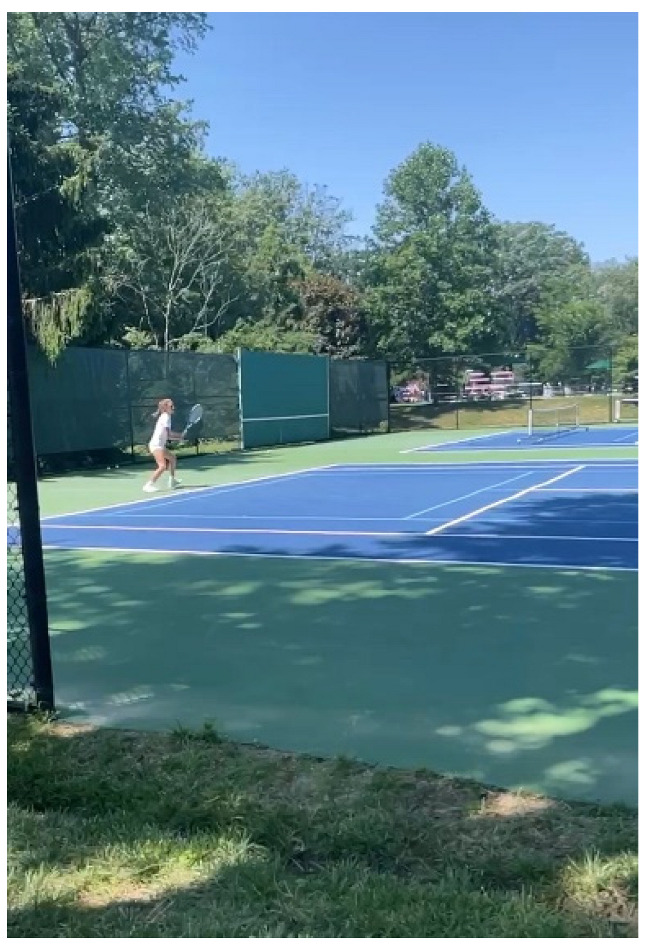
An outdoor tennis court among trees.

Another participant shared a photo of an outdoor tennis match (Figure 4). During the discussion, she shared that she had always enjoyed playing tennis outside but had not connected that being among the trees and grass of nature contributed to her enjoyment and stress relief.

*“By playing a game I cherish outside in the open, I am able to let go of all the stress I am feeling. By finding an activity you enjoy doing while being outside, your five senses are triggered while you hear the peaceful birds in the background while hitting a floating yellow ball. You feel a sense of comfort and peace as you relax as time flies. When you go home for the day, you are ready to take on a new adventure because your body has reset itself.”*

#### 3.2.3. Theme 3: Nature Gives Us Space to Find Solutions

**Figure 5:**
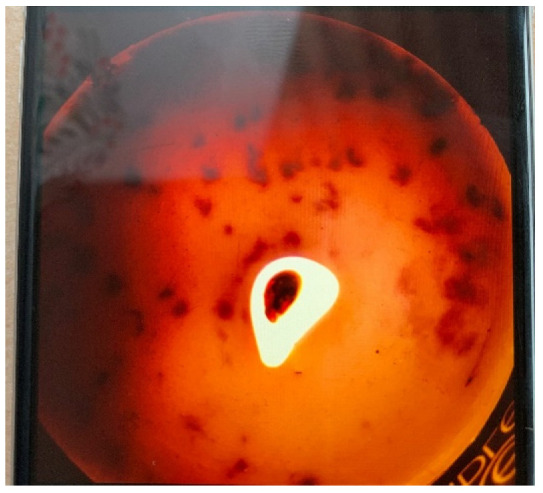
A lit candle, shown from above.

A powerful example of the need for space was shared by an adolescent who lives with many family members in a small urban dwelling. Her photograph (Figure 5) shows a burning candle, taken from above, to represent her need for space. She shared that she finds that space by going to greenspaces in her neighborhood.

*“Space is a tool that not everyone has enough of, but nature provides it to us.”*

**Figure 6:**
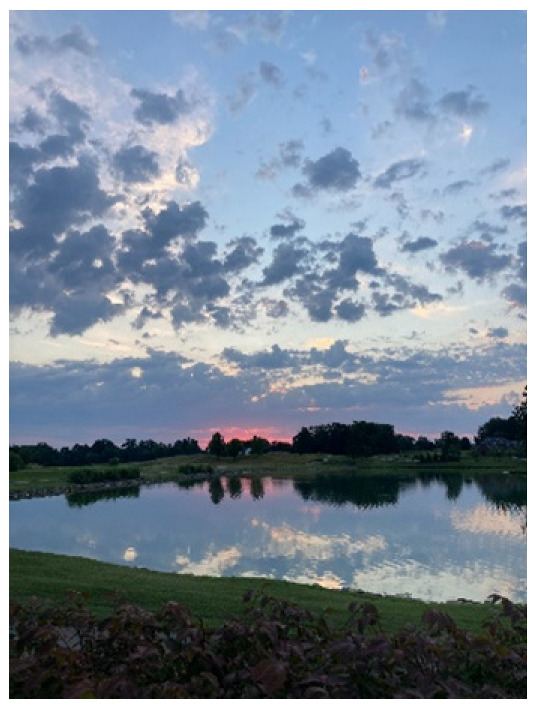
A pond.

**Figure 7:**
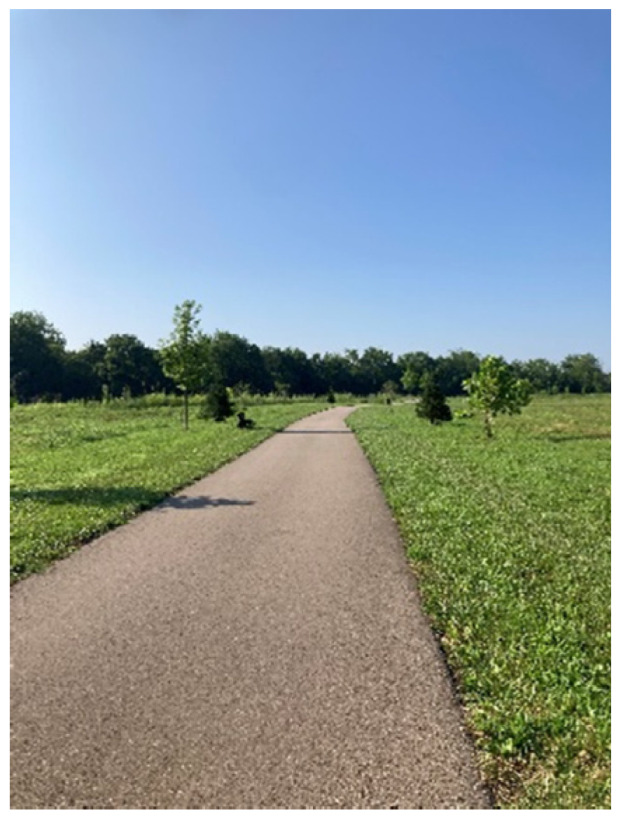
A paved walking path.

Two participants shared photos of open green spaces, one a pond at a golf course (Figure 6) and the other a paved path (Figure 7). Interestingly, the photo of the paved path was originally presented with negative connotations, with the participant sharing that she did not perceive as much benefit from man-made or cultivated nature versus wild nature. However, after group discussion, that photograph became representative of the need for space.

*“Space is sometimes needed to be able to think clearly or just take a breather.”*

*“Nature provides an environment free of stress and its ever-expanding existence. It takes away pressure for finding solutions and provides silence away from people and our everyday lives.”*

#### 3.2.4. Theme 4: We Want to Find Time to Enjoy Nature

**Figure 8:**
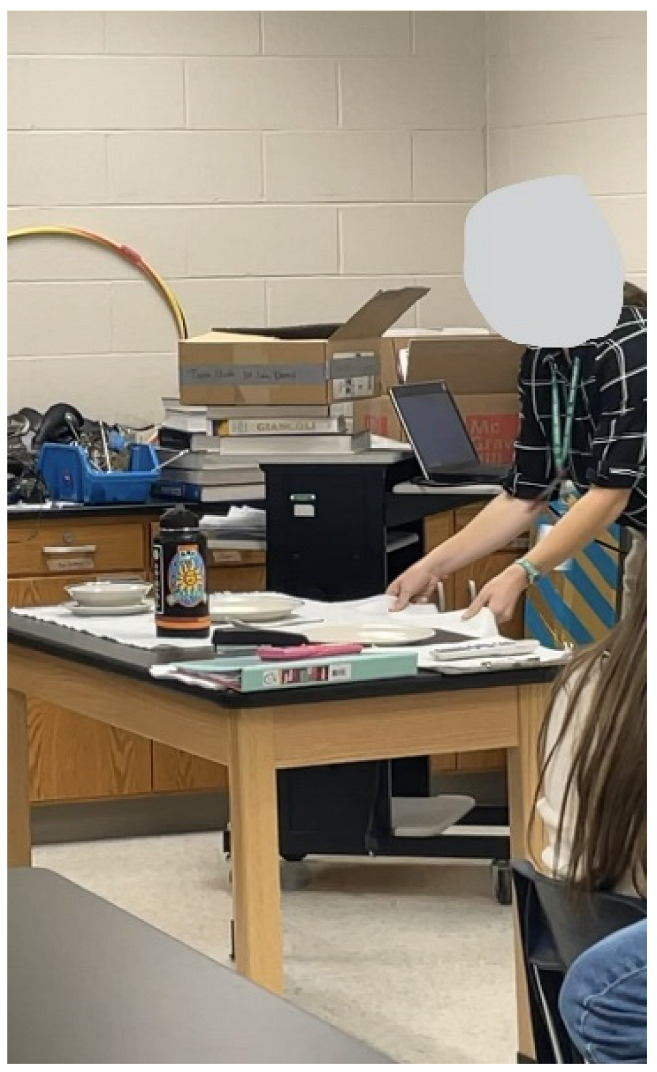
An indoor classroom without windows.

**Figure 9:**
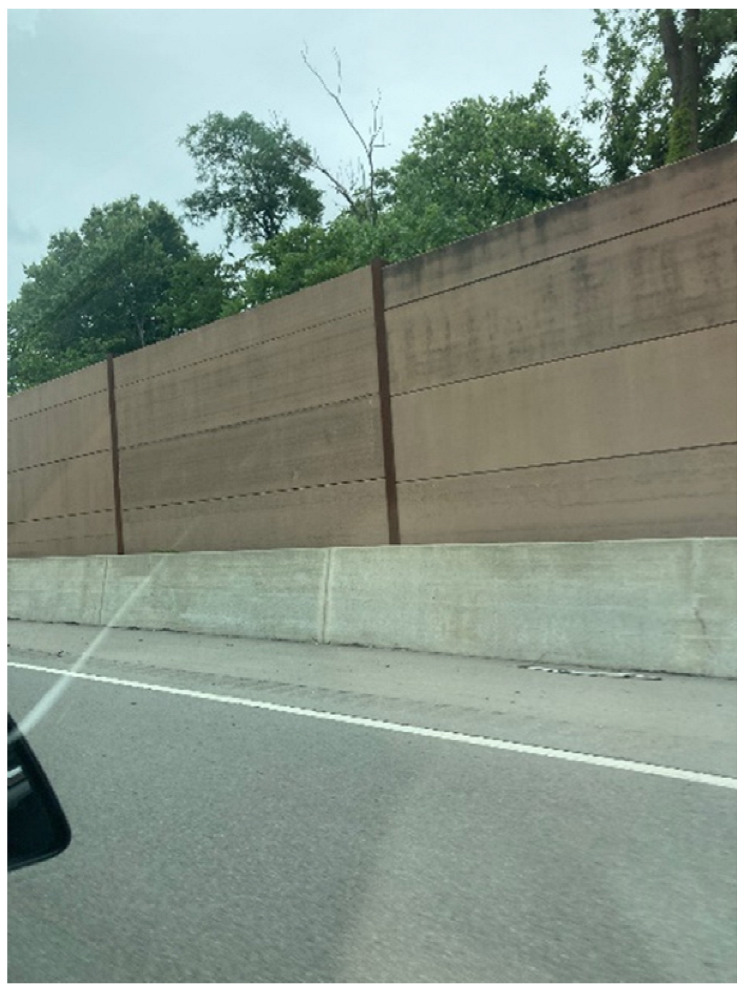
A highway view.

**Figure 10:**
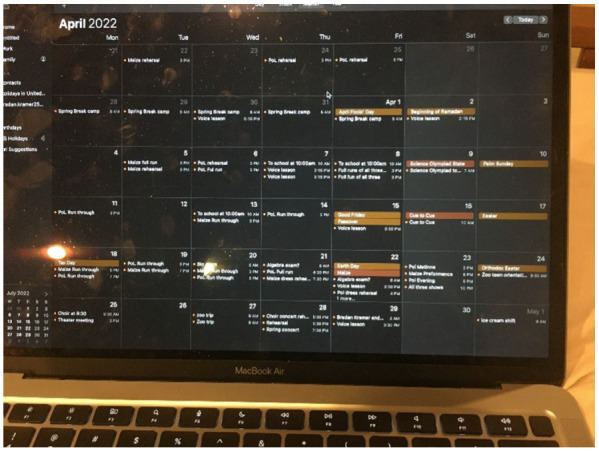
One participant’s schedule, not including the school day.

Participants provided examples of barriers they experience to finding time for nature. Participating youth shared photographs within this theme depicting a teacher in a classroom without windows (Figure 8), a highway view from a car windwo (Figure 9), and a screenshot of one participant’s calendar (Figure 10). These photographs represented the lack of nature where youth spent time (namely in school and in driving long distances between home, school, and activities) and the lack of available time to seek nature.

*“Our daily lives can become very hectic and distracting and we can forget to take time to relax and decompress. When we take time to seek out peace in nature, we can find relief from stress and have a higher quality of life.”*

### 3.3. Community Exhibition—Feedback from Youth Participants

As a participatory research method, a key component of Photovoice methodology is a community exhibition where participants present findings and advocate for change. This was a powerful experience for our youth participants. They led the session, presenting their photographs and the meaning they conveyed through the images. During the question and answer panel discussion, adolescents shared their feelings about the value of the research process:

*“(It was) enlightening that nature is everywhere and other people have the same thoughts that I do…”*

*“I was inspired to spend more time connecting with nature”*

*“I sought the tools I need to cope with stress and anxiety.”*

*“This is going to help a lot of people. Nature can help a lot of people.”*

*“(It was) encouraging… talking and sharing our experiences (without) fear of judgement.”*

## 4. Discussion

### 4.1. Discussion of Findings

Previously, we defined nature generally as trees, flowers, plants, and grasses; this was contrasted with built or man-made surroundings. It is important to recognize that even this general definition does not encompass the meaning of the term “nature” for everyone. Additionally, we recognize that exposure to nature is not a positive experience for everyone as nature can be associated with concerns for personal safety. For our study, we spent time in our first group session discussing the definition of nature with our participants. This was a necessary and effective way to build consensus around the term’s definition and how it would be used for the duration of our study.

Through deeper understanding of how adolescents perceive and use the nature around them to relieve stress, our findings are relevant for families, educators, students, healthcare professionals, and anyone who works with or cares for adolescents. This project helped to amplify the voices of these adolescents and provided them with a venue for engagement and leadership as advocates for positive change. As volunteers in a program aimed at engaging adolescents in conservation, we anticipated that our participants would experience more positively toward nature than a general population of adolescents, which should foster ecological behaviors such as seeking nature to relieve stress [16]. We were somewhat surprised that this was generally, but not always, the case. While these adolescents had a strong affinity for nature and were often intentional in seeking time in nature (for example, going to a park or walking in the woods), they were not always cognizant of using nature to relieve their stress. Through the group process, participants came to identify that nature can be a powerful tool for actively reducing stress and supporting their mental health. It may be that personal preference for an environment amplifies the beneficial effects of that environment [6,17]. While these participants demonstrated a preference for nature, its utility in decreasing stress was not always intentional. Given this finding, future research should investigate whether the intentional use of nature, rather than passive exposure, strengthens associations between nature and mental health.

Our participants recognized through the photovoice process that nature can be found all around them. One participant illustrated this by sharing that the stress he was experiencing related to his job was lessened when he entered to the patio and noticed a pot of colorful flowers. Another participant expressed that she experienced less stress during an overstimulating car ride when she noticed a bird in a tree outside her car window among a developed streetscape. This finding suggests that awareness of nature may also amplify its benefits for relieving stress. Adolescents may benefit from consistent reminders that nature can be beneficial for reducing stress and generally supporting their mental health.

The finding that seemed to resonate the most with our participants was Theme 4: We Want to Find Time to Enjoy Nature. There was much agreement among the group when a youth shared that he felt that his schedule of academic and social obligations, while enjoyable, did not leave time for him to seek nature. Participants then identified that as they learn to balance competing demands, they may need to be reminded to spend time in nature and perhaps think creatively about how their scheduled activities may occur with nature incorporated into them. These youth participants indicated that they looked to the adults in their lives to help them create a schedule that allows for their own wellness but noted that oftentimes those adults have similar trouble doing so for themselves.

Our findings support that adolescents can use nature to relieve their stress, and nature is an important tool for supporting adolescent mental health. We assert that all adolescents should have the opportunity to experience the benefits of nature. As natural landscapes are developed to make way for human expansion, it is important to protect and expand natural habitats. This is especially important in urban areas where nature may already be lacking. Ensuring the availability of nature is a key component of environmental justice initiatives that seek to eliminate the historical inequities in the distribution of resources [18], including nature such as parks, preserves, and greenspaces.

### 4.2. Methodological Considerations

We found photovoice methodology to be a useful tool in our investigation. Using photographs allowed youth participants to easily share ideas and begin open dialogue. At the conclusion of the project, participants shared their gratitude for having this safe space for camaraderie with their peers and to feel valued and not judged for the thoughts and opinions that they shared.

### 4.3. Strengths and Limitations

The major strength of this investigation was the participating youth who provided expert insight into their experiences of stress and nature. These findings simply would not be possible without their willingness to openly share among the group.

Our goal was to more deeply understand youth participants’ personal experiences in using nature to relieve their stress. As dictated by our research question, our use of qualitative methodology and enrolling eight participants for group discussion is a strength of our study. However, due to the relative lack of diversity in terms of sociodemographic factors, our findings may only offer limited insights for the experiences of youth residing in the urban core or primarily rural communities, youth with diagnosed mental health disorders, or other age groups such as young children or adults.

A limitation of our study may be that we conducted this research in summer, which in our Midwestern climate may allow greater interaction with nature. In investigations of nature and health, the effects of seasonality continue to be difficult to parse.

### 4.4. Suggestions for Future Research

Our findings are important for informing future quantitative, qualitative, and mixed-methods research. This study may be replicated with other populations, including rural-dwelling adolescents and those with clinical diagnoses relevant to stress such as anxiety, to establish transferability. Quantitative researchers may consider using geotagging to better understand where, when, and how often adolescents visit natural places to relieve their stress. Additionally, our study may provide qualitative findings for an exploratory sequential mixed-methods study to investigate the relationships between nature and stress.

## 5. Conclusions

This qualitative participatory study used photovoice methodology to understand how nature relieves stress among conservation-engaged adolescents. Youth participants agreed that nature is a tool that can be used to decrease their stress. However, even those who seek out nature and profess enjoyment of nature may not consciously or explicitly recognize their use of nature as a tool for relieving stress. This may be important for the psychosocial benefits of nature in relieving stress and is an important area for future research.

In consideration of the practical implications of our findings, we offer the following recommendations for how nature can be leveraged as a tool for decreasing stress in the critical life stage of adolescence:

1. Caregivers, educators, psychologists, social workers, nurses, physicians, and anyone who works with or cares for adolescents should include nature when teaching skills for stress management.
2. Adult caregivers should teach and model for adolescents the skills to maintain a balanced schedule that allows time for wellness, and adolescents should be regularly encouraged to find time to spend in nature.
3. Adolescents who use nature to relieve stress can help their peers recognize that nature can be a tool to decrease their own stress.
4. Nature should be creatively woven into adolescents’ regularly scheduled activities. For example, nature can be incorporated into schools through outdoor classrooms, learning gardens, and by intentionally planting trees in the sightline of classroom windows.
5. Future research should continue to investigate the physiological and psychological benefits of time in nature. Investigations of the roles of intentionality and awareness in the use of nature to relieve stress may contribute to our understanding of these relationships.
6. Future research should ascertain whether our findings resonate with different populations. Suggestions include adolescents who do not feel connected to nature, those in the urban core or rural communities, other geographic areas, clinical adolescent populations, and other age populations.
7. Adolescent programs should consider offering meaningful group interactions between peers in a collaborative, supportive, and non-judgmental environment.

## Figures and Tables

**Table 1:** Description of photovoice sessions.

Session	Timeframe	Activities
1	2 h	Introduce the study and photovoice to the study participants.
		Build community through introducing youth participants to each other and defining “nature”.
2	2 h	Using the SHOWeD method, discuss Prompt 1: “What nature is around you (home, school, work, other places you spend time)? How does nature make you feel? What is it about nature that can help relieve your stress?”
3	2 h	Using the SHOWeD method, Discuss Prompt 2: “Last week, we talked about how you sometimes use nature to relieve your stress. What might prevent you from doing so?”
4	2 h	Review themes with study members (member checking) and identify which themes best fit each image.
5	1 h	Present themes to community through a gallery walk poster session and a moderated Q&A session.

## Data Availability

All data related to this study are stored on the secure institutional OneDrive system. Data may be made available by contacting the lead author.
